# Infant feeding practices among mildly wasted children: a retrospective study on Nias Island, Indonesia

**DOI:** 10.1186/1746-4358-7-3

**Published:** 2012-03-21

**Authors:** Dyah Ayu Inayati, Veronika Scherbaum, Ratna Chrismiari Purwestri, Elizabeth Hormann, Nia Novita Wirawan, Julia Suryantan, Susan Hartono, Maurice Alexander Bloem, Rosnani Verba Pangaribuan, Hans Konrad Biesalski, Volker Hoffmann, Anne Camilla Bellows

**Affiliations:** 1 Institute for Social Sciences in Agriculture, Center Gender and Nutrition (430b), University Hohenheim, Fruwirthstrasse 14-16, 70593 Stuttgart, Germany; 2 The European Institute for Breastfeeding and Lactation, Kramsach, Austria; 3 Faculty of Medicine, Study Program Nutrition, University of Brawijaya, Malang, Indonesia; 4 Church World Service, Jakarta, Indonesia; 5 Church World Service, New York, USA; 6 (Former) SEAMEO-TROPMED Regional Centre for Community Nutrition, Jakarta, Indonesia; 7 Institute for Biological Chemistry and Nutrition, University Hohenheim, Stuttgart, Germany

**Keywords:** Breastfeeding, Complementary foods, Infant feeding, Wasted children, Nias Island

## Abstract

**Background:**

This study investigated the infant feeding practices of participating mothers who were recruited into a research project aimed at improving the nutritional status of mildly wasted children (< -1.0 to ≥ -1.5 Weight-for-Height Z*-score*s) aged ≥ 6 to < 60 months on Nias Island, Indonesia.

**Methods:**

Cross-sectional, questionnaire-based interview of mothers of the index children (*n *= 215) who were admitted to the community program for mildly wasted children in the study area. Four focus groups and twenty in-depth interviews were conducted to explore further information on infant feeding practices in the study area.

**Results:**

Retrospective results indicated that 6% of the mothers never breastfed. Fifty two percent of mothers initiated breastfeeding within six hours of birth, but 17% discarded colostrum. Exclusive breastfeeding until 6 months of age was practiced by 12%. Seventy-four percent of the mothers offered supplementary liquids besides breast milk within the first 7 days of life, and 14% of infants received these supplementary liquids from 7 days onwards until 6 months of age. Moreover, 79% of the infants were given complementary foods (solid, semi-solid, or soft foods) before 6 months of age. About 9% of the children were breastfed at least two years. Less than one in five of the mildly wasted children (19%) were breastfed on admission to the community program. Qualitative assessments found that inappropriate infant feeding practices were strongly influenced by traditional beliefs of the mothers and paternal grandmothers in the study areas.

**Conclusion:**

Generally, suboptimal infant feeding was widely practiced among mothers of mildly wasted children in the study area on Nias Island, Indonesia. To promote breastfeeding practices among mothers on Nias Island, appropriate nutrition training for community workers and health-nutrition officers is needed to improve relevant counseling skills. In addition, encouraging public nutrition education that promotes breastfeeding, taking into account social-cultural factors such as the influence of paternal grandmothers on infant feeding practice, is needed.

## Background

Delayed breastfeeding initiation, colostrum deprivation, supplementary feeding of breast milk substitutes, early introduction of complementary feeding, and incorrect weaning from breast milk are commonly found practices in communities around the world [1-5]. Nationwide data in Indonesia has shown that only 39% of infants experience early initiation of breastfeeding (within one hour after birth) and 32% are exclusively breastfed for less than six months [6]. Seventy-five percent of children aged 6 to 9 months were continuing to be breastfed and received complementary foods [6]. A prior survey by the Church World Service (CWS) on Nias Island, North Sumatra Province documented that solid foods were introduced as early as 3 months and appropriate breastfeeding practices were uncommon (CWS Indonesia unpublished report).

It is well known that breastfeeding influences a child's health positively and improves nutritional status [7-9]. A meta-analysis from three developing countries showed that infants who were not breastfed had a six-fold greater risk of dying from infectious diseases within the first two months of life than those who were breastfed [10]. Six months of exclusive breastfeeding and continued breastfeeding in the first year of life could also prevent 1.3 million child deaths worldwide according to systematic reviews from the Bellagio Child Survival Study Group [11]. In addition, incorrect infant feeding practices pose significant risks for malnutrition among children under the age of five [1,12,13].

To date, no data has been collected describing the infant feeding patterns among mothers of mildly wasted children on Nias Island. This Nias study tried to assess the history of infant feeding practices among respondents who were recruited into the community program for mildly wasted children on Nias Island. In addition, the study tried to determine the need for enhanced educational intervention to improve the infant feeding situation there. Data were collected via a structured questionnaire that was completed in face-to-face interviews as well as in focus group discussions.

## Methods

### Study setting and population

The study was conducted from October 2007 to September 2008 in the Church World Service (CWS) project area in Gunung Sitoli, Sirombu, and Mandrehe Districts, Nias Island, North Sumatra Province, Indonesia. A total of 215 mothers of mildly wasted children (< -1.0 to ≥ -1.5 Weight-for-Height-Z*-score*s, according to WHO/NCHS reference data [14]) aged ≥ 6 to < 60 months participated in the study, which aimed at improving the nutritional status of children under five years of age in this community [15].

All mothers were interviewed at admission and were informed about the purpose of the study. The interviews were conducted by health-nutrition officers of the implementing partners (CWS) who had attended a two-day practical training course on interview skills prior to the actual data collection. Informed consent was sought prior to the interview, following the protocols set by the 1995 Helsinki Declaration, as revised in Edinburgh 2000. The study was approved by the Ethical Committee of the Faculty of Medicine, University of Brawijaya (Nr. 25/PEPK/VIII/2007).

### Breastfeeding definitions

Exclusive breastfeeding is defined as an infant being fed solely breast milk (including his mother's expressed milk or milk from a wet nurse) and nothing else, not even water, with the exception of prescribed medicines and vitamin supplements [12]. Timely breastfeeding initiation is referred to as the start of breastfeeding within one hour after delivery [12]. The discontinuation of breastfeeding before six months is defined as 'early' discontinuation of breastfeeding according to the breastfeeding recommendations of the Ministry of Health, Republic of Indonesia.

### Data collection

The structured questionnaire used for interviewing was pre-tested for cultural sensitivity before data collection. The questionnaire forms were completed during face-to-face interviews with the mothers. Data collected included demographic and anthropometric variables, infant feeding practices and history.

Qualitative data assessments were performed to explore further information on infant feeding practices in the study area. The data were collected in the course of four focus group interviews, and during individual in-depth interviews. Focus group interviews were conducted in four villages in the study area (*n *= 23). In addition, twenty in-depth interviews were done with selected caregivers whose children were recruited into the study. A structured questionnaire was pre-tested prior to the study to improve comprehension. To overcome the possible cultural, literacy, and language barriers, one experienced health and nutrition officer of the implementing partner (with similar ethnicity) was present during the qualitative data assessments. With respect to focus group interviews, the moderators were trained in data collection techniques prior to the qualitative data assessments.

### Data analysis

Data were coded and analyzed using the PASW/SPSS package version 19.0 (SPSS Inc., Chicago, IL, USA). Values were presented in percentages and means (± standard deviation). Qualitative data was audio taped and transcribed verbatim by an experienced transcriber. Interviewers and researchers reviewed the transcripts at least once to confirm the accuracy of the transcribed statements. The data were further evaluated via thematic analysis and coded for themes.

## Results

### Socio-demographic characteristics

Two hundred and fifteen mothers and their children, assessed as mildly wasted, were eligible for further analysis. Table 1 shows selected socio-demographic characteristics of the study population. Of 215 eligible children, approximately 44% of those admitted to the study were girls. The highest proportion (28%) of them was found to be between ≥ 24 to < 36 months. The average Weight-for-Height (WHZ) score was -1.3 ± 0.1, whereas the average Height-for-Age (HAZ) score was -1.5 ± 1.5 and Weight-for-Age (WAZ) was -1.9 ± 0.8.

**Table 1:** Selected characteristics of the study population on Nias Island (*n *= 215)

	**Frequency (%**)
**Female children**, n (%)	95 (44.2)
**Age group (months)**, n (%)	
6 - < 12	13 (6.0)
≥ 12 - < 24	40 (18.6)
≥ 24 - < 36	61 (28.4)
≥ 36 - < 48	42 (19.5)
≥ 48-60	59 (27.4)
**Weight-for-Height-z-score **(mean ± SD)	-1.3 ± 0.1
**Height-for Age-z-score **(mean ± SD)	-1.5 ± 1.5
**Weight-for-Age-z-score **(mean ± SD)	-1.9 ± 0.8
**Age group (< 30 y)**, n (%)	
Mothers	140 (65.1)
Fathers	89 (41.4)
**Occupation (farmer)**, n(%)	
Mothers	149 (69.3)
Fathers	122 (56.7)
**Education of caregiver (≥ 6 y)**, n(%)	
Mothers	80 (37.2)
Fathers	123 (57.2)
Paternal grandmothers	99 (46.0)
**Household asset: ownership of TV**, n(%)	54 (25.1)
**Time use for income generating activities **(mean ± SD)	6.4 ± 2.6
**Time use for child caring **(mean ± SD)	2.1 ± 1.3

The majority of the mothers (65%) were under 30 years old, had less than six years of schooling (63%), and worked mainly as farmers (69%). Mothers were involved in income generating activities for an average of six hours per day, while two hours were dedicated to child care activities.

All families selected for analysis comprised two-parent households. More than 40% of the fathers were < 30 years and worked in the agricultural sector (57%). Fathers had the best educational level (57% of them went to school for more than six years), followed by paternal grandmothers (46%), and mothers (37%).

The majority of respondent households did not have a television (75%).

### Infant feeding practices at admission time

**Figure 1:**
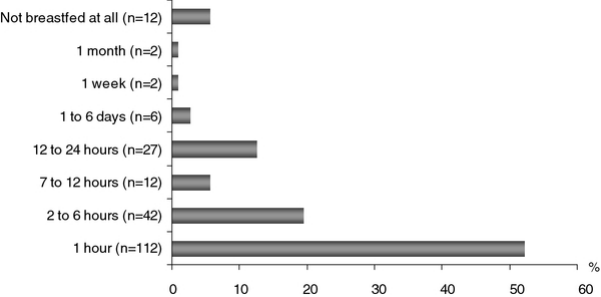
**Time of breastfeeding initiation after birth among respondents Six percent (*n *= 12) of the children were not breastfed at all**. According to the mothers, reasons for not offering breast milk to their children immediately after delivery included *"the breast milk did not come out due to inverted nipples *(*n *= 4)", "*mother was suffering from sore nipples after childbirth *(*n *= 2)", "*child did not want to suck the nipples *(*n *= 4)", and "*mother was sick after childbirth *(*n *= 2)". As a substitute for colostrum, mothers gave infant formula (*n *= 4), sugar water (*n *= 7) or sweet tea (*n *= 1) after childbirth. In addition to feeding breast milk substitutes, these mothers introduced complementary foods very early (≤ 1 month of age)

Almost 48% of the mothers did not start to breastfeed their babies within the first hour after delivery, as recommended by WHO/UNICEF. However, the majority of respondents (72%) initiated breastfeeding within the first six hours after birth (Figure 1).

The practice of giving early supplementary liquids was also frequently found in the study area. Seventy-four percent of mothers reported that they introduced liquid foods besides breast milk to their infants during the first seven days of life (data not shown). Another 14% of infants were introduced to supplementary liquids between ≥ 8 days and < 6 months. Infant formula (32%) and tea (26%) were the preferred supplementary liquids mentioned by the mothers in the study area.

Although WHO/UNICEF recommends exclusive breastfeeding for the first six months of life, only 12% of mildly wasted children (*n *= 25) in the study area were exclusively breastfed during the first six months of their lives.

**Figure 2:**
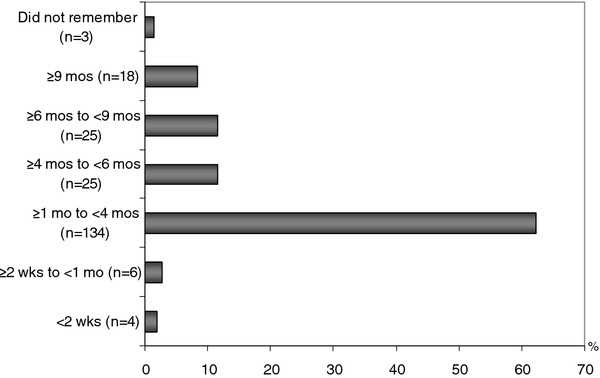
**Age of introducing solid, semi-solid or soft foods among mothers of mildly wasted children (*n *= 215) To determine the point prevalence of breastfeeding, mothers were asked whether they had still been breastfeeding their children in the last 24 hours before admission (Table 2)**. The majority of children in the study area were no longer breastfed at the time of admission (76%). The main reasons for discontinuing breastfeeding included: child was too old to be breastfed, breast refusal, a new pregnancy, infant or maternal illness, as well as decreased breast milk production.

Seventy-nine percent (*n *= 169) of the mothers introduced complementary foods (solid, semi-solid or soft foods) earlier than the international recommendation of 6 months (Figure 2). One hundred and forty-four out of 215 caregivers (67%) had already introduced complementary foods before the children were four months old. Five percent of the respondents (*n *= 10) even offered complementary foods when the children were less than one month old. Only 12% of the mothers introduced complementary foods to their children after six months of age. Rice porridge (*n *= 138), milk porridge (*n *= 31), boiled steamed rice (*n *= 30), fruits (*n *= 7), and commercially prepared baby foods (*n *= 6) were commonly reported as the first foods given to the babies in the study area.

With respect to the reported duration of breastfeeding, most mothers (30%) breastfed their children for a total of 6 to 12 months (*n *= 49), followed by 25% and 24% who breastfed for ≥ 4 and < 6 months and ≥ 12 and < 24 months respectively. Thirteen mothers stopped breastfeeding before the babies were four months old, but we also found that 16 respondents continued breastfeeding for more than two years (Table 2).

**Table 2:** Breastfeeding prevalence at admission time among mothers of mildly wasted children (*n *= 215)

	n = 215
**Continuing breastfeeding, n (%)**	40 (18.6%)

Age of children (months)	
≥ 6 - < 12	5 out of 13
≥ 12 - < 24	17 out of 40
≥ 24 - < 60	18 out of 162
**Stopped breastfeeding, n (%)**	163 (75.8%)

Reported duration of breastfeeding practice in months n (%)	**n = 163**
< 4	13 (7.8%)
≥ 4 - < 6	41 (25.2%)
≥ 6 - < 12	49 (30.1%)
≥ 12 - < 24	39 (23.9%)
≥ 24	16 (9.9%)
Did not remember breastfeeding duration	5 (3.1%)
**Never breastfed, n (%)**	12 (5.6%)

## Discussion

### Breastfeeding initiation

In our study on Nias, we found that more than half of the mothers (52%) initiated breastfeeding (*la femenu*) in the first hour after delivery. In total, 72% of the mothers breastfed their newborns in the first six hours after baby's birth This finding is in line with previous studies in Timor Leste, India, and Turkey [16-18]. However, 28% of the Nias mothers initiated breastfeeding six hours after birth or even later, and 17% discarded the colostrum due to local traditional beliefs similar to the ones in the study in India [19,20]. Reasons given for not feeding colostrum to newborns included the traditional belief that colostrum was "dirty (*n *= 24), cheesy (*n *= 14), and indigestible (*n *= 8)" and that "children will suffer from stomach ache/*afökhö talu *(*n *= 21)", "children will get illnesses such as fever/*mofa'aukhu *(*n *= 15)", and "children will be stupid/*bodo *(*n *= 4)".

The notion that "colostrum is dirty" (*n *= 24) is likely due to the yellowish color of colostrum that is much different from the whitish color of mature mother's milk. The yellow color of colostrum was identified with 'dirty (*ta'unö*)' breast milk, while the white color of mature milk was believed to be 'clean' maternal milk. Colostrum was considered by some mothers to be 'cheesy (*oŵoyu*)' breast milk that had been produced late in the mother's pregnancy (*n *= 14) and had no nutritional value (*n *= 10). Colostrum was also believed to have a bitter taste/*afeto *(*n *= 12). Thus several mothers considered colostrum to be an inappropriate food for the newborn and believed it would be harmful to the infant's health.

"According to my mother-in-law and other senior female relatives, colostrum is not a healthy food for the newborns. This first milk is dirty and cheesy. If it is given to the newborn, my infant will suffer from stomach ache . . ." (Mother in Bawadesolo)

The practice of discarding colostrum is strongly rooted in the study area. We argue that the strong role of senior family members, particularly senior women such as the paternal grandmother, in prohibiting the consumption of colostrum for newborns has sustained this custom, and that it has been carried out for generations in the study area.

It is known that early initiation of breastfeeding will result in better establishment of breastfeeding practices, including longer duration of breastfeeding. This practice also ensures that newborns receive food with high nutritional value at the beginning of their lives. Early breastfeeding initiation can protect the newborn from potentially harmful pre-lacteal feeding practices which deprive the infant of the important immune-protective properties of colostrum [12].

### Practice of pre-lacteal feeding and use of supplementary liquids

Wide use of pre-lacteal foods has been identified from previous studies in India, Ethiopia, Bangladesh, and Tanzania [17,20-24]. The practice of pre-lacteal feeding was also common in our study area (personal information with Health and Nutrition officers CWS). Sugar water (*n *= 37) and infant formula (*n *= 25) were the two most preferred pre-lacteal liquids mentioned by the mothers. Several mothers believed that providing pre-lacteal liquids could help the newborns to resist hunger (*n *= 30). They perceived that because of the process of childbirth, the newborns experience fatigue, which then leads to feelings of hunger. Therefore, supplementary feeding serves the perception that this practice reduces infants' hunger in the first hours of their life.

Although colostrum is enough to sustain the nutritional needs of newborn babies without any additional foods [12], this concept was not widely known in the study area. Giving pre-lacteal foods (e.g. formula milk or anything else other than breast milk) delays the production of breast milk [25,26]. If infants consume pre-lacteal foods or supplementary foods, this interferes with suckling at the breast, the release of prolactin and, ultimately, the production of the mother's milk [12]. This leads to lower breast milk production and a shorter period of breastfeeding [27,28]. Pre-lacteal foods are often a source of newborn infections and diarrhea [12,17]. The delay of breastfeeding initiation and giving pre-lacteal feeds creates a vicious cycle. The practice of pre-lacteal feeding delays the initiation of breastfeeding and the delay in initiating breastfeeding promotes further pre-lacteal feeding.

This Nias study found that three out of four children received supplementary feeding (liquids) in the first seven days of their lives. The common reason for giving supplementary feeding in the study area was perceived breast milk insufficiency (*n *= 22), which was likely due to the delay of breastfeeding initiation. Bearing in mind that the frequency and the duration of breastfeeding are important factors in stimulating breast milk production, it is not surprising that incorrect breastfeeding practice such as providing supplementary foods results in insufficient production of breast milk.

### Introduction of complementary foods (solid, semi-solid or soft foods)

The appropriate time for introducing complementary foods and the types of foods are crucial factors to be considered in appropriate infant and child feeding practices. According to Agudo et al. [4], findings from several studies showed that the majority of mothers in developing countries initiated complementary feeding too early. Other studies have had similar findings [29-31]. Our study also revealed that early introduction of solid and semi-solid foods was common in the study area.

Most of our respondents preferred to introduce complementary foods (solid, semi-solid, or soft food) when the children were one to four months old. This was in line with the results of our qualitative assessments that found that the majority of mothers believed that an appropriate time for introducing complementary foods was between one and four months (*n *= 32).

A perceived decline in the production of breast milk was the main reason mentioned for early introduction of complementary foods in the study area (*n *= 30). Practice of pre-lacteal feeding, skepticism about the sufficiency of their own breast milk production, inappropriate time of introduction of complementary foods, as well as infrequent and too short duration of breastfeeds, were apparently responsible for the decline in the maternal milk supply. On the other hand, perceived insufficient breast milk production also led to too early initiation of complementary feeding. In both situations, mothers believed that their child's crying was a signal of insufficient food intake. Thus, the provision of complementary foods seemed to them to be the only way to satisfy the child.

*"Mothers in this area have usually introduced complementary foods when infants are four months old. However, they will provide complementary foods earlier if the child still cries although breast milk has already been given" *(Mother in Tugala Gawu)

The opinions of senior female members of the family, especially the paternal grandmother (*ina matua*), about the infant's well-being played an important role in the decision of mothers to introduce solid, semi-solid, or soft foods (*n *= 23). If an infant was considered by the female in-law to be too 'thin' (*afuo*), a mother was likely to initiate complementary foods. As reported by respondents, the decision to provide complementary foods was usually not accompanied by maintaining breastfeeding frequency. It is therefore not surprising that increasing supplemental food was associated with less breastfeeding and breast milk production declined. Even worse, breastfeeding was generally no longer practiced after a child was given complementary foods. This is a very common pattern around the world reflecting a misunderstanding of complementary feeding (solids) as a replacement for breastfeeding, rather than a complement to it.

Early introduction of liquids and solids is unnecessary and unwise because it can reduce the duration and frequency of breastfeeding [12,24], as previously observed in our study area. Premature introduction of complementary foods increases the risk of infant morbidity and mortality [12,24]. We therefore conclude that children in our Nias study area face increased and unnecessary health risks due to inappropriate infant feeding practices.

### The use of infant formula

Commercially produced infant formula was provided as a supplementary food (liquid) in addition to breast milk during the first seven days of life (32%), as a supplementary food in addition to breastfeeding within six months (13%), or to fully replace breast milk (2%) in the study area. Some mothers perceived that infant formula had highly valuable nutrients that were as good as, or even better than breast milk (*n *= 10). Therefore, they believed that if the household had the financial means, formula should be provided to the babies. However, according to Gribble et al. [32] and Mulder-Baalbergen et al. [33], several considerations should be taken into account when providing infant formula at the household level, particularly in emergency situations. These include the availability of clean water, energy for heating/boiling water and sterilizing bottles and other equipment, household finances as well as the ability of caregivers to prepare infant formula correctly according to the manufacturer's instruction [32,33]. These requirements appeared to be very difficult for respondents in our study area to meet.

As stated in WHO/FAO guidelines [34], preparing infant formula according to the manufacturer's instructions is still not sufficient and safe because of the inherent bacterial contamination in many powdered formulas. Manufacturers do not usually give accurate information about an adequate temperature for the water to be mixed with the powdered formula. In order to destroy the bacteria, water must be boiled and then cooled to no less than 70°c (between 70° and 90°C) before mixing. This was also not known in the study area. Accurate assessment of water temperature is also highly uncertain.

### Exclusive breastfeeding practice

In our study area, only one out of nine mothers practiced exclusive breastfeeding with their infants. This low prevalence of exclusive breastfeeding has been commonly reported in studies in India, Bangladesh, Sri Lanka, Turkey, and Tanzania [18,20,23,24,30]. Based on qualitative assessment, the most common barrier to practicing exclusive breastfeeding in the study area was perceived insufficient breast milk production (*n *= 30). Other reasons, such as believing that the child was starving (*n *= 15), the mother's activity outside her home (*n *= 10), poor knowledge of the benefits of exclusive breastfeeding (*n *= 7), and the mother's illness (*n *= 7) were also mentioned by several mothers.

A mother may make a decision to add supplemental fluids or soft complementary foods at too early an age if she is feeling discouraged about her milk production. If a mother believes that she cannot produce a sufficient quantity of breast milk, she tends to decrease her breastfeeding frequency. This will result in decreasing breast milk production.

*"I am sure that I did not have sufficient breast milk production. My breast is too small and I am too thin as well. My mother-in-law said that this child (pointed out her two-year old son), was too thin when he was an infant, although I had already given my breast milk to him. Therefore she said to me that I should give him other foods as he was three months old." *(Mother in Tögideu)

Infrequent and brief feeds are commonly practiced among mothers in the study area (personal communication with Health and Nutrition Officers CWS Nias). These two factors combined were likely to be main contributors to low breast milk production. This 'insufficient breast milk syndrome' has led to the belief that mother's milk could not provide sufficient nutrients for the infants. Thus, most of the mothers in the study area believed that a six month period of exclusive breastfeeding could endanger the health and nutritional status of their young children. Indeed, this would be the case, if babies were not fed frequently enough.

Brief feeds are actually very common in some regions where mothers are constantly active doing manual agricultural work or gathering water or fuel. When these mothers carry their babies with them (mostly on their backs), they may compensate for the short feeds by a more frequent feeding pattern.

In addition to the constraints identified above, we surmise that nutrition education on Nias Island might not be optimal with regard to promoting appropriate breastfeeding practices, and might be contributing to the low prevalence of exclusive breastfeeding in the first six months in our study area. Based on Agampodi et al. [30], a major determinant of exclusive breastfeeding practices is the health care provider's knowledge about, attitudes towards, and skills for promoting exclusive breastfeeding. Therefore, we speculate that insufficient breastfeeding-related knowledge among health personnel and community workers may have reinforced mothers' and families' perceptions that have led to the low prevalence of exclusive breastfeeding rates in our study area. With reference to communication to Health and Nutrition Officers of CWS Nias, it may, for example, be that the policy change of 2004 that adjusted the recommendations for exclusive breastfeeding from "four months" to "six months" had not reached the grass roots level of public health staff on Nias Island - even almost a decade after this policy change.

### Breastfeeding prevalence at admission

Breastfeeding prevalence among respondents was low at admission into the study program for mildly wasted children on Nias Island. One of the reasons was probably related to the high proportion of children who were ≥ 24 months (75%). Another reason was presumably related to the heavy workloads of mothers in the study area. We found that women in this study had to perform income-generating activities for an average of six hours per day in informal sectors. Most of these activities took place away from their homes. Therefore, it would likely be difficult to breastfeed during their absence from home. This was usually followed by a decline of breastfeeding frequency and, as previously stated, a decrease in breast milk production and, ultimately, cessation of breastfeeding. However, such constraints can be prevented if the mothers have proper information on how to maintain milk production while separated from the baby, and how to compensate for the absence with more frequent breastfeeds when mother and child are together. A six hour separation is not an insurmountable barrier to exclusive breastfeeding.

We also found several cultural factors influencing the decision to cease breastfeeding. In our study area, the perception that prolonged breastfeeding would interfere with the child's growth is widespread. Several mothers believed that children who breastfed until the age of two years or beyond would favor consuming breast milk instead of family foods (*n *= 23). They believed that the child's daily nutrient intake would not meet the recommended levels if consumption of home-based family food was too low, and this would lead to the impaired growth of children (personal communications with several caregivers). We argue that the concern that children will not eat family foods properly if they are still breastfed is essentially a child-rearing issue. Parents can and should set reasonable limits on fulfilling children's consumption wishes beyond infancy. Parents need to understand that the way feeding is organized is a management question, and for optimal infant feeding the consumption of family foods should take place alongside of continued breastfeeding during a child's first two years.

The belief that pregnant women should not breastfeed their children was also a factor hindering the practice of breastfeeding during pregnancy (*n *= 15). According to Nias culture, breastfeeding by a pregnant mother could harm the fetus because it would decrease the fetus' food intake (personal communication with traditional healers in Sirombu, Nias). They also claimed that a pregnant mother who persisted in breastfeeding would deliver a thin (*afuo*), sickly (*mofökhö-fökhö*), and feeble-minded (*bodo*) newborn baby. Therefore, most of the pregnant mothers in our study area did not breastfeed their young children. Considering that short birth spacing was commonly found in the study area, a new pregnancy would lead to a decision to wean too early. This probably contributed to the low breastfeeding rate in our study.

The belief that a mother who is ill should not breastfeed her child was widespread in the study area. According to local beliefs, the illness of a mother is transmitted to the breastfed child through breast milk. Therefore, some mothers in our study area did not breastfeed their children when they were ill (*n *= 9). In this situation, mothers preferred to provide family foods to the young children and halted breastfeeding during the illness. Unfortunately, the elimination of breastfeeding practices affects the breast milk production. A decreased breast milk supply was usually followed by breastfeeding cessation. This may also have contributed to the low breastfeeding prevalence in our study area.

### The importance of appropriate educational programs

Bearing in mind that the protective effect of breastfeeding is especially significant in populations with high infant mortality, low literacy, poor sanitation facilities, poor nutritional status and generally low economic status [23], accurate information on the importance of early initiation of breastfeeding, six months of exclusive breastfeeding, and continued breastfeeding after the introduction of complementary foods would be particularly important for our respondents in this Nias study who likely faced similar living conditions. Therefore, the promotion of correct breastfeeding practices should be high on the agenda for any health-nutrition activities aimed to improve the well-being of young children on Nias Island.

It is also important to motivate mothers to practice exclusive breastfeeding for six months with continued breastfeeding until two years or beyond, as well as introducing timely complementary feeding (six months), since these are high-priority infant feeding indicators for child survival [35].

Appropriate infant feeding promotion would help prevent faulty feeding practices, such as the provision of pre-lacteal and supplementary foods (liquids) and too early introduction of complementary foods. These inappropriate infant feeding practices increase the risk of illness, malnutrition, and even death among infants and young children [13].

Special attention to appropriate breastfeeding and complementary feeding interventions should be given to the mothers/caregivers of young infants of the ages most likely to be affected by malnutrition. This susceptible period is when young infants are introduced to foods and liquids other than breast milk. Focusing educational programs on this target group is likely to be more cost-effective than interventions that include a wider range of mothers/caregivers [13].

Our qualitative assessments also found that the support of family members was an important influencing factor for improving infant feeding practices in the study area. We learned that paternal grandmothers have great influence on infant/child feeding decisions, such as duration of exclusive breastfeeding, and the time to introduce complementary feeding [15], as was also observed by Aubel in the Grandmother Project [36]. Therefore any interventions aimed at improving young child feeding practices, particularly infant feeding behaviors, should also target paternal grandmothers or partners of pregnant women, and the families of newborn infants, especially those members who play an important role in caring for and feeding young children. Grandmothers, who were reported in our study to have a negative influence on breastfeeding, should be included in programs for breastfeeding promotion. Other target groups such as community workers, health professionals, and traditional birth attendants should also be given the necessary guidance, appropriate training, and support with respect to breastfeeding promotion.

## Conclusion

In summary, the present study revealed suboptimal infant child feeding practices among the mothers of mildly wasted children in the study area on Nias Island, Indonesia. Thus, appropriate educational interventions on breastfeeding and complementary feeding are important in order to improve existing infant feeding practices, as well as reduce the risk of malnutrition in young children. These educational strategies should focus particularly on counteracting various myths related to infant feeding practices in the area targeted. To promote breastfeeding practices, it is vital to improve the counseling skills of community workers, as well as related breastfeeding campaign actors in the study area. Moreover, breastfeeding promotion in the community should target not only maternal caregivers, but also other family members, particularly husbands and paternal grandmothers, taking into account the social and cultural situation on Nias Island.

## Competing interests

The authors declare that they have no competing interests.

## Authors' contributions

DAI and VS designed/implemented the study and drafted the manuscript; DAI, RCP, NNW and RVP organized and supervised the study in the field; EH, MAB, JS, SH, VH, HKB and ACB helped with the study design, gave valuable comments and contributed to the final version of the manuscript. All authors read and approved the final manuscript.
